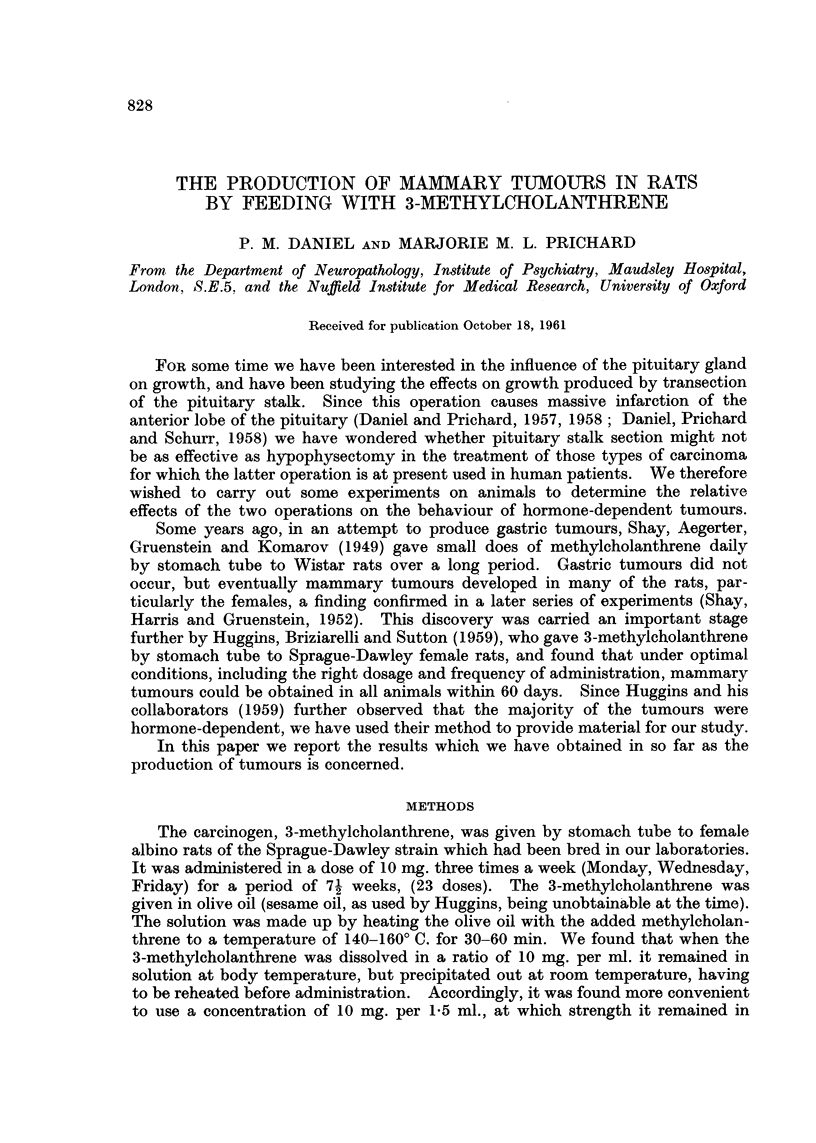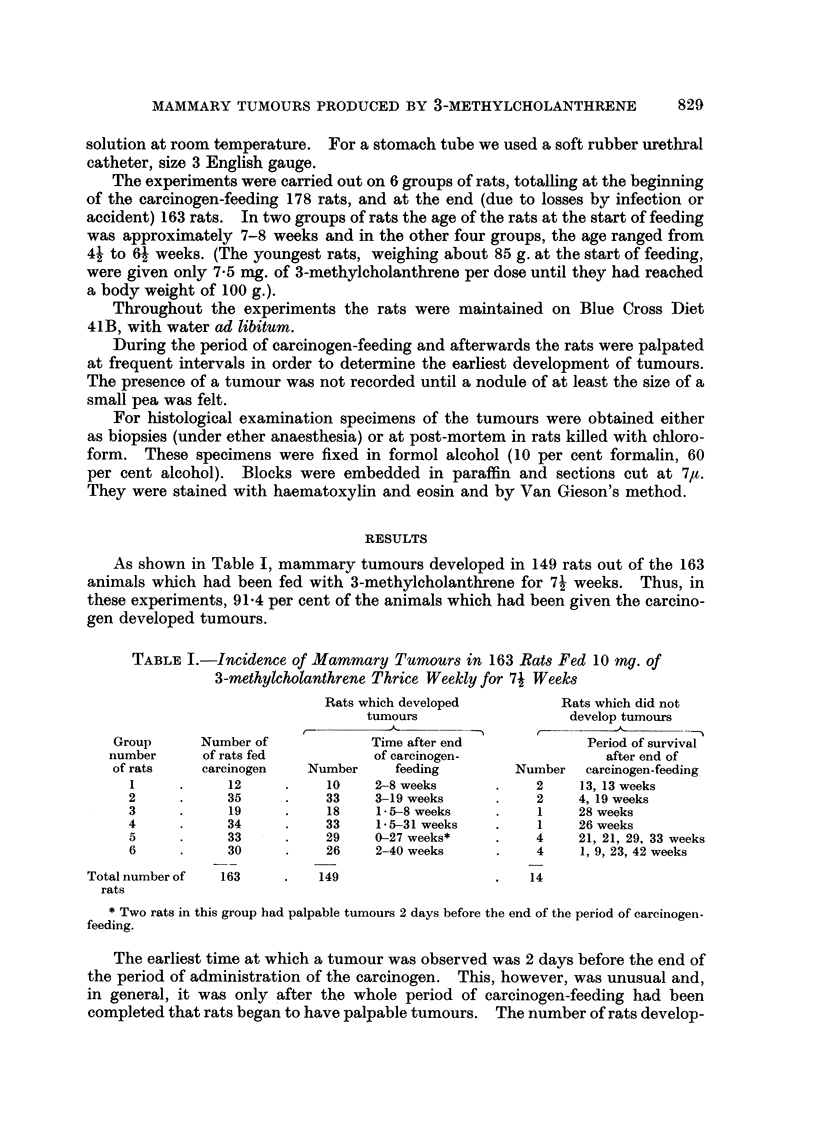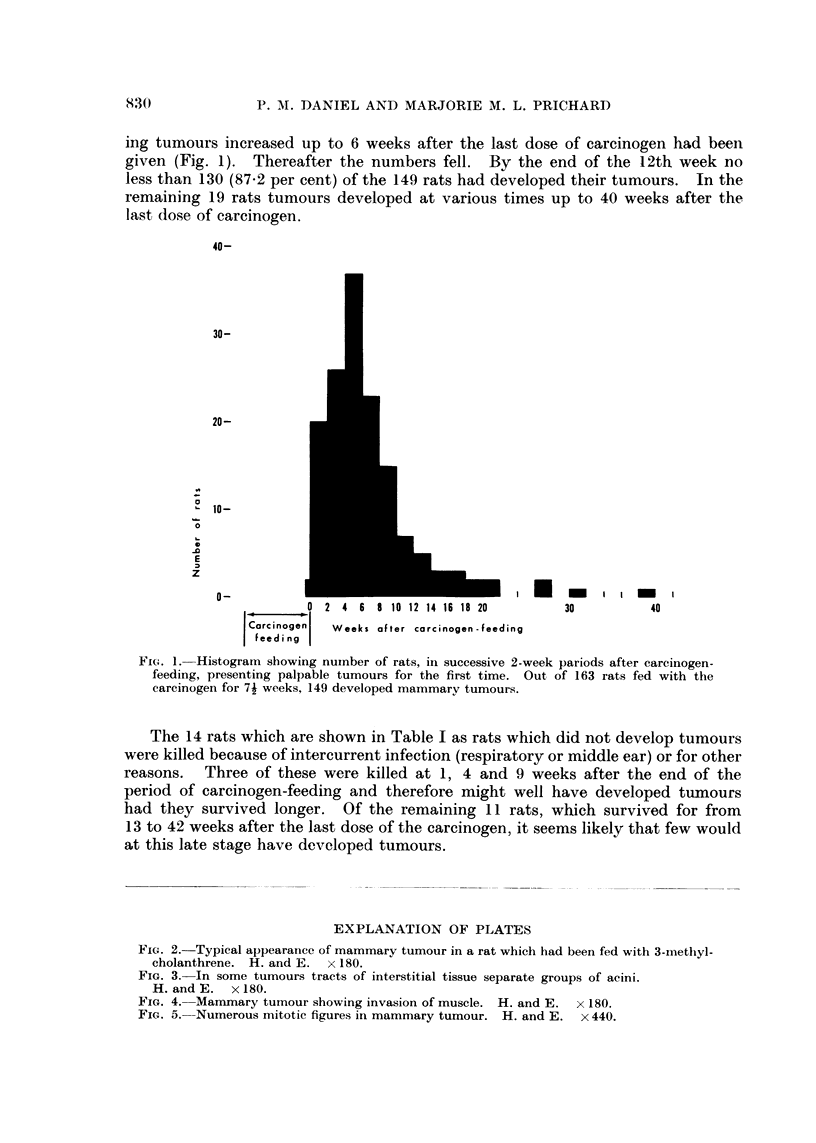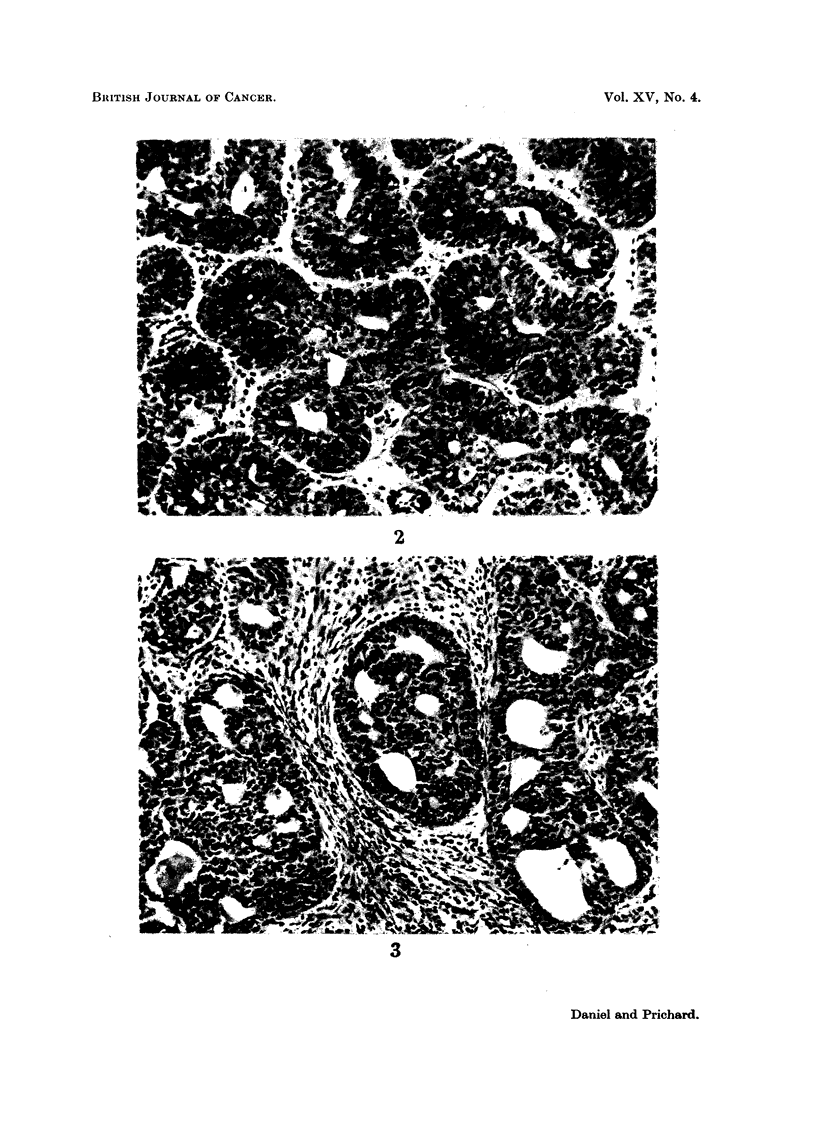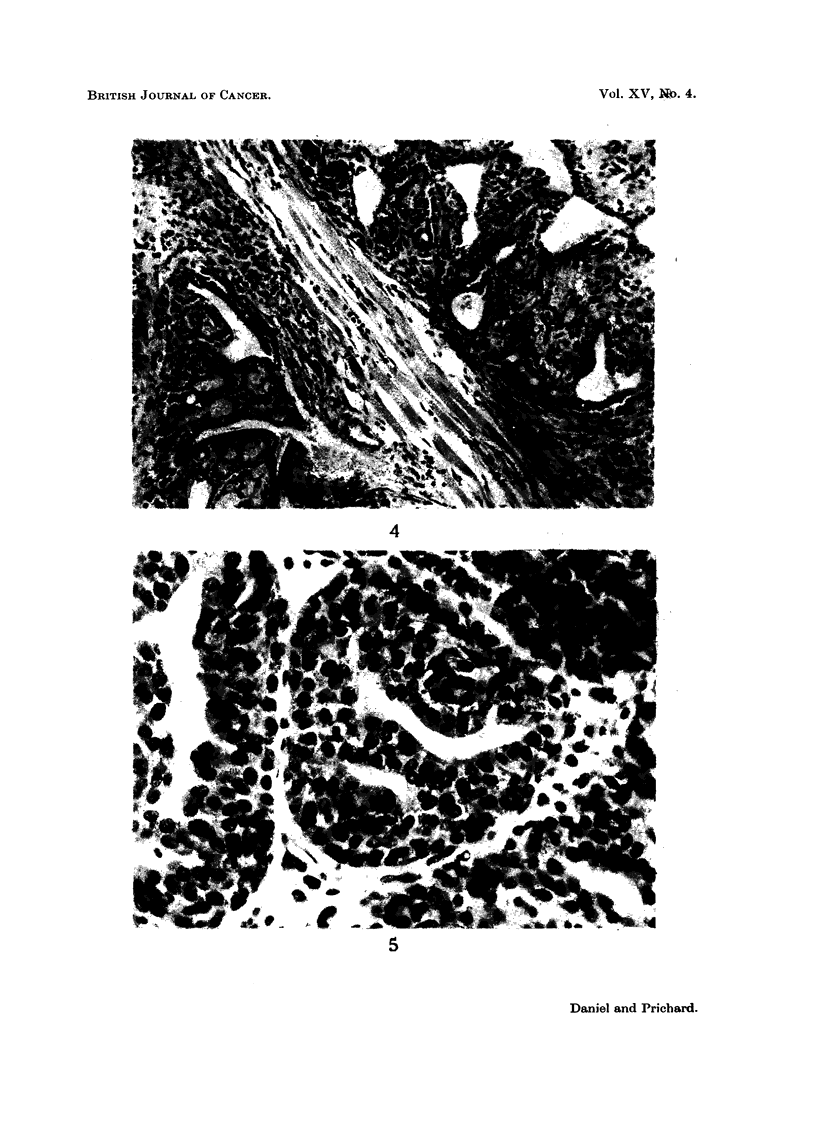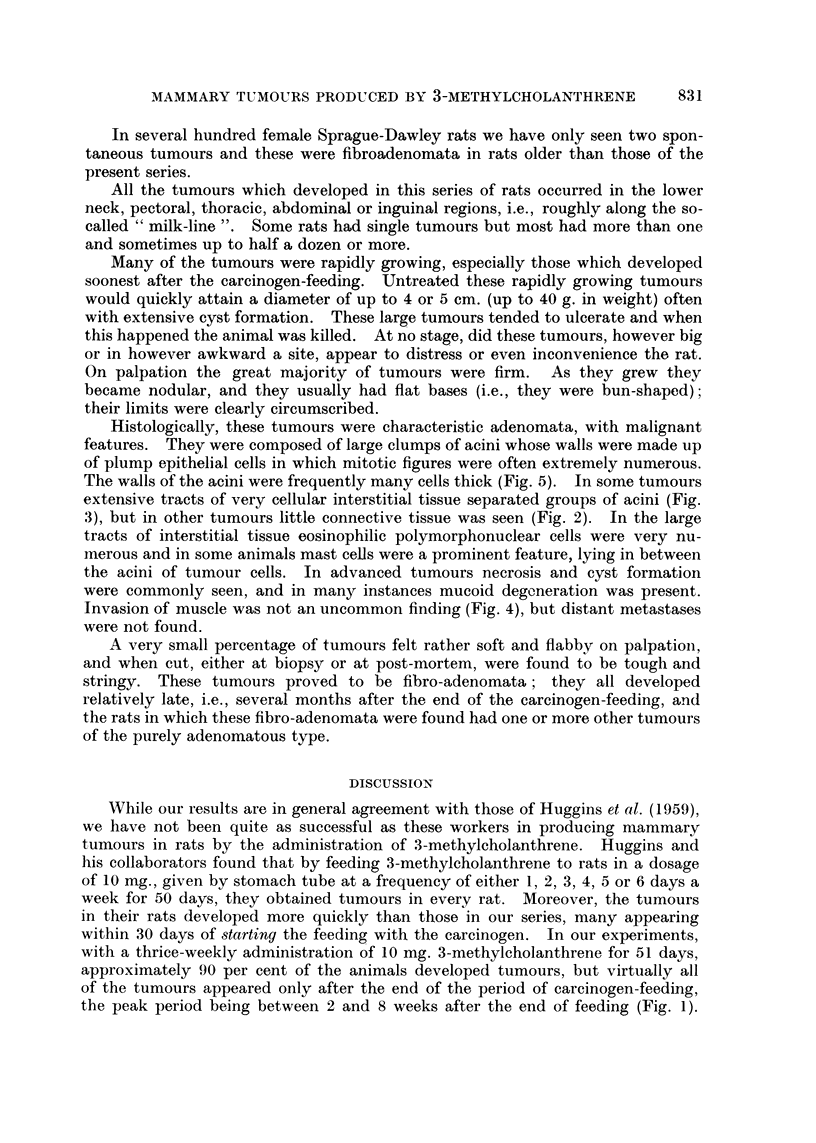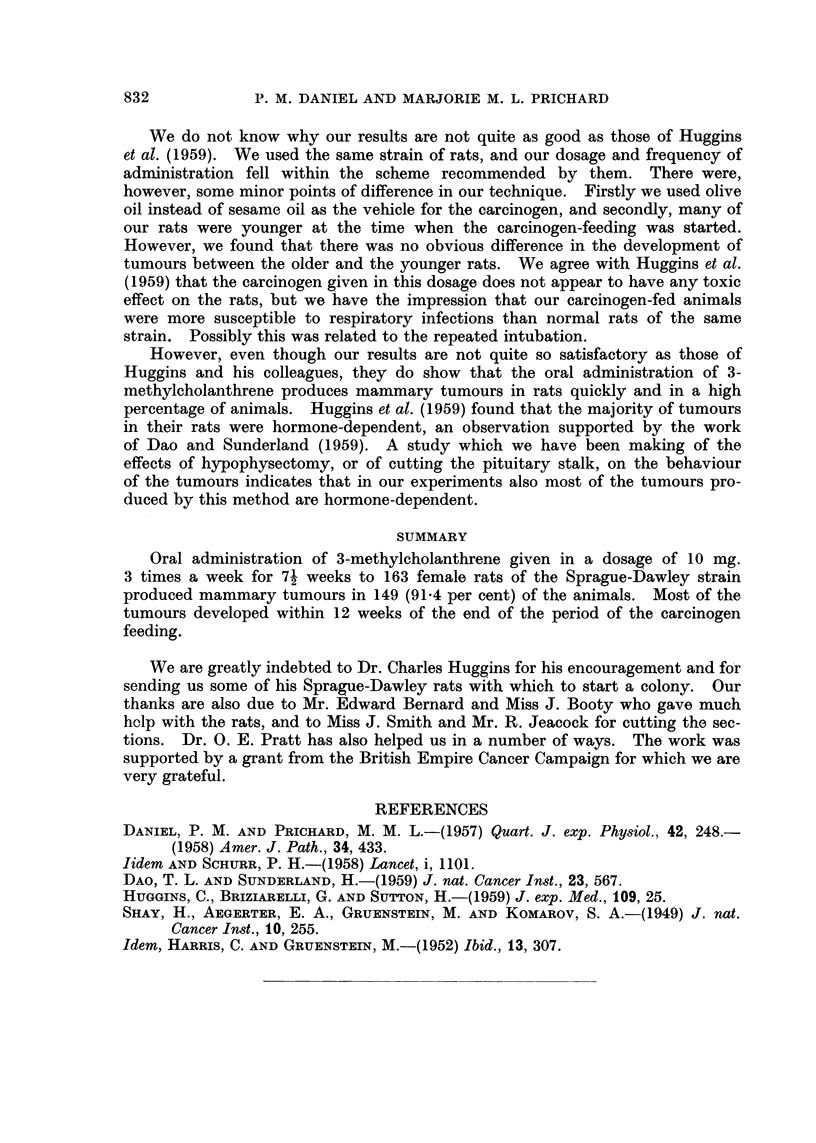# The Production of Mammary Tumours in Rats by Feeding with 3-Methylcholanthrene

**DOI:** 10.1038/bjc.1961.95

**Published:** 1961-12

**Authors:** P. M. Daniel, Majorie M. L. Prichard

## Abstract

**Images:**


### THE PRODUCTION OF MAMMARY TUMOURS IN RATS BY FEEDING WITH 3-METHYLCHOLANTHRENE

P. M. DANIEL AND MARJORIE M. L. PRICHARD

*From the Department of Neuropathology, Institute of Psychiatry, Maudsley Hospital, London, S.E.5, and the Nuffield Institute for Medical Research, University of Oxford*

Received for publication October 18, 1961

FOR some time we have been interested in the influence of the pituitary gland on growth, and have been studying the effects on growth produced by transection of the pituitary stalk. Since this operation causes massive infarction of the anterior lobe of the pituitary (Daniel and Prichard, 1957, 1958 ; Daniel, Prichard and Schurr, 1958) we have wondered whether pituitary stalk section might not be as effective as hypophysectomy in the treatment of those types of carcinoma for which the latter operation is at present used in human patients. We therefore wished to carry out some experiments on animals to determine the relative effects of the two operations on the behaviour of hormone-dependent tumours.

Some years ago, in an attempt to produce gastric tumours, Shay, Aegerter, Gruenstein and Komarov (1949) gave small does of methylcholanthrene daily by stomach tube to Wistar rats over a long period. Gastric tumours did not occur, but eventually mammary tumours developed in many of the rats, particularly the females, a finding confirmed in a later series of experiments (Shay, Harris and Gruenstein, 1952). This discovery was carried an important stage further by Huggins, Briziarelli and Sutton (1959), who gave 3-methylcholanthrene by stomach tube to Sprague-Dawley female rats, and found that under optimal conditions, including the right dosage and frequency of administration, mammary tumours could be obtained in all animals within 60 days. Since Huggins and his collaborators (1959) further observed that the majority of the tumours were hormone-dependent, we have used their method to provide material for our study.

In this paper we report the results which we have obtained in so far as the production of tumours is concerned.

### METHODS

The carcinogen, 3-methylcholanthrene, was given by stomach tube to female albino rats of the Sprague-Dawley strain which had been bred in our laboratories. It was administered in a dose of 10 mg. three times a week (Monday, Wednesday, Friday) for a period of $7\frac{1}{2}$ weeks, (23 doses). The 3-methylcholanthrene was given in olive oil (sesame oil, as used by Huggins, being unobtainable at the time). The solution was made up by heating the olive oil with the added methylcholanthrene to a temperature of 140–160° C. for 30–60 min. We found that when the 3-methylcholanthrene was dissolved in a ratio of 10 mg. per ml. it remained in solution at body temperature, but precipitated out at room temperature, having to be reheated before administration. Accordingly, it was found more convenient to use a concentration of 10 mg. per 1·5 ml., at which strength it remained in solution at room temperature. For a stomach tube we used a soft rubber urethral catheter, size 3 English gauge.

The experiments were carried out on 6 groups of rats, totalling at the beginning of the carcinogen-feeding 178 rats, and at the end (due to losses by infection or accident) 163 rats. In two groups of rats the age of the rats at the start of feeding was approximately 7–8 weeks and in the other four groups, the age ranged from 4½ to 6½ weeks. (The youngest rats, weighing about 85 g. at the start of feeding, were given only 7·5 mg. of 3-methylcholanthrene per dose until they had reached a body weight of 100 g.).

Throughout the experiments the rats were maintained on Blue Cross Diet 41B, with water *ad libitum.*

During the period of carcinogen-feeding and afterwards the rats were palpated at frequent intervals in order to determine the earliest development of tumours. The presence of a tumour was not recorded until a nodule of at least the size of a small pea was felt.

For histological examination specimens of the tumours were obtained either as biopsies (under ether anaesthesia) or at post-mortem in rats killed with chloroform. These specimens were fixed in formol alcohol (10 per cent formalin, 60 per cent alcohol). Blocks were embedded in paraffin and sections cut at 7$\mu$. They were stained with haematoxylin and eosin and by Van Gieson's method.

### RESULTS

As shown in Table I, mammary tumours developed in 149 rats out of the 163 animals which had been fed with 3-methylcholanthrene for 7½ weeks. Thus, in these experiments, 91·4 per cent of the animals which had been given the carcinogen developed tumours.

TABLE I.—*Incidence of Mammary Tumours in* 163 *Rats Fed* 10 *mg. of* 3-*methylcholanthrene Thrice Weekly for* 7½ *Weeks*

| | | Rats which developed tumours | | Rats which did not develop tumours | |
|---|---|---|---|---|---|
| Group number of rats | Number of of rats fed carcinogen | Number | Time after end of carcinogen-feeding | Number | Period of survival after end of carcinogen-feeding |
| 1 | 12 | 10 | 2–8 weeks | 2 | 13, 13 weeks |
| 2 | 35 | 33 | 3–19 weeks | 2 | 4, 19 weeks |
| 3 | 19 | 18 | 1·5–8 weeks | 1 | 28 weeks |
| 4 | 34 | 33 | 1·5–31 weeks | 1 | 26 weeks |
| 5 | 33 | 29 | 0–27 weeks* | 4 | 21, 21, 29, 33 weeks |
| 6 | 30 | 26 | 2–40 weeks | 4 | 1, 9, 23, 42 weeks |
| Total number of rats | 163 | 149 | | 14 | |

* Two rats in this group had palpable tumours 2 days before the end of the period of carcinogen-feeding.

The earliest time at which a tumour was observed was 2 days before the end of the period of administration of the carcinogen. This, however, was unusual and, in general, it was only after the whole period of carcinogen-feeding had been completed that rats began to have palpable tumours. The number of rats developing tumours increased up to 6 weeks after the last dose of carcinogen had been given (Fig. 1). Thereafter the numbers fell. By the end of the 12th week no less than 130 (87·2 per cent) of the 149 rats had developed their tumours. In the remaining 19 rats tumours developed at various times up to 40 weeks after the last dose of carcinogen.

Fig. 1.—Histogram showing number of rats, in successive 2-week pariods after carcinogen-feeding, presenting palpable tumours for the first time. Out of 163 rats fed with the carcinogen for 7½ weeks, 149 developed mammary tumours.

The 14 rats which are shown in Table I as rats which did not develop tumours were killed because of intercurrent infection (respiratory or middle ear) or for other reasons. Three of these were killed at 1, 4 and 9 weeks after the end of the period of carcinogen-feeding and therefore might well have developed tumours had they survived longer. Of the remaining 11 rats, which survived for from 13 to 42 weeks after the last dose of the carcinogen, it seems likely that few would at this late stage have developed tumours.

---

EXPLANATION OF PLATES

Fig. 2.—Typical appearance of mammary tumour in a rat which had been fed with 3-methylcholanthrene. H. and E. ×180.

Fig. 3.—In some tumours tracts of interstitial tissue separate groups of acini. H. and E. ×180.

Fig. 4.—Mammary tumour showing invasion of muscle. H. and E. ×180.

Fig. 5.—Numerous mitotic figures in mammary tumour. H. and E. ×440.

2

3

Daniel and Prichard.

4

5

Daniel and Prichard.

In several hundred female Sprague-Dawley rats we have only seen two spontaneous tumours and these were fibroadenomata in rats older than those of the present series.

All the tumours which developed in this series of rats occurred in the lower neck, pectoral, thoracic, abdominal or inguinal regions, i.e., roughly along the so-called " milk-line ". Some rats had single tumours but most had more than one and sometimes up to half a dozen or more.

Many of the tumours were rapidly growing, especially those which developed soonest after the carcinogen-feeding. Untreated these rapidly growing tumours would quickly attain a diameter of up to 4 or 5 cm. (up to 40 g. in weight) often with extensive cyst formation. These large tumours tended to ulcerate and when this happened the animal was killed. At no stage, did these tumours, however big or in however awkward a site, appear to distress or even inconvenience the rat. On palpation the great majority of tumours were firm. As they grew they became nodular, and they usually had flat bases (i.e., they were bun-shaped); their limits were clearly circumscribed.

Histologically, these tumours were characteristic adenomata, with malignant features. They were composed of large clumps of acini whose walls were made up of plump epithelial cells in which mitotic figures were often extremely numerous. The walls of the acini were frequently many cells thick (Fig. 5). In some tumours extensive tracts of very cellular interstitial tissue separated groups of acini (Fig. 3), but in other tumours little connective tissue was seen (Fig. 2). In the large tracts of interstitial tissue eosinophilic polymorphonuclear cells were very numerous and in some animals mast cells were a prominent feature, lying in between the acini of tumour cells. In advanced tumours necrosis and cyst formation were commonly seen, and in many instances mucoid degeneration was present. Invasion of muscle was not an uncommon finding (Fig. 4), but distant metastases were not found.

A very small percentage of tumours felt rather soft and flabby on palpation, and when cut, either at biopsy or at post-mortem, were found to be tough and stringy. These tumours proved to be fibro-adenomata ; they all developed relatively late, i.e., several months after the end of the carcinogen-feeding, and the rats in which these fibro-adenomata were found had one or more other tumours of the purely adenomatous type.

### DISCUSSION

While our results are in general agreement with those of Huggins *et al.* (1959), we have not been quite as successful as these workers in producing mammary tumours in rats by the administration of 3-methylcholanthrene. Huggins and his collaborators found that by feeding 3-methylcholanthrene to rats in a dosage of 10 mg., given by stomach tube at a frequency of either 1, 2, 3, 4, 5 or 6 days a week for 50 days, they obtained tumours in every rat. Moreover, the tumours in their rats developed more quickly than those in our series, many appearing within 30 days of *starting* the feeding with the carcinogen. In our experiments, with a thrice-weekly administration of 10 mg. 3-methylcholanthrene for 51 days, approximately 90 per cent of the animals developed tumours, but virtually all of the tumours appeared only after the end of the period of carcinogen-feeding, the peak period being between 2 and 8 weeks after the end of feeding (Fig. 1).

We do not know why our results are not quite as good as those of Huggins *et al.* (1959). We used the same strain of rats, and our dosage and frequency of administration fell within the scheme recommended by them. There were, however, some minor points of difference in our technique. Firstly we used olive oil instead of sesame oil as the vehicle for the carcinogen, and secondly, many of our rats were younger at the time when the carcinogen-feeding was started. However, we found that there was no obvious difference in the development of tumours between the older and the younger rats. We agree with Huggins *et al.* (1959) that the carcinogen given in this dosage does not appear to have any toxic effect on the rats, but we have the impression that our carcinogen-fed animals were more susceptible to respiratory infections than normal rats of the same strain. Possibly this was related to the repeated intubation.

However, even though our results are not quite so satisfactory as those of Huggins and his colleagues, they do show that the oral administration of 3-methylcholanthrene produces mammary tumours in rats quickly and in a high percentage of animals. Huggins *et al.* (1959) found that the majority of tumours in their rats were hormone-dependent, an observation supported by the work of Dao and Sunderland (1959). A study which we have been making of the effects of hypophysectomy, or of cutting the pituitary stalk, on the behaviour of the tumours indicates that in our experiments also most of the tumours produced by this method are hormone-dependent.

SUMMARY

Oral administration of 3-methylcholanthrene given in a dosage of 10 mg. 3 times a week for 7½ weeks to 163 female rats of the Sprague-Dawley strain produced mammary tumours in 149 (91·4 per cent) of the animals. Most of the tumours developed within 12 weeks of the end of the period of the carcinogen feeding.

We are greatly indebted to Dr. Charles Huggins for his encouragement and for sending us some of his Sprague-Dawley rats with which to start a colony. Our thanks are also due to Mr. Edward Bernard and Miss J. Booty who gave much help with the rats, and to Miss J. Smith and Mr. R. Jeacock for cutting the sections. Dr. O. E. Pratt has also helped us in a number of ways. The work was supported by a grant from the British Empire Cancer Campaign for which we are very grateful.

### REFERENCES

DANIEL, P. M. AND PRICHARD, M. M. L.—(1957) *Quart. J. exp. Physiol.*, **42**, 248.—(1958) *Amer. J. Path.*, **34**, 433.

*Iidem* AND SCHURR, P. H.—(1958) *Lancet*, i, 1101.

DAO, T. L. AND SUNDERLAND, H.—(1959) *J. nat. Cancer Inst.*, **23**, 567.

HUGGINS, C., BRIZIARELLI, G. AND SUTTON, H.—(1959) *J. exp. Med.*, **109**, 25.

SHAY, H., AEGERTER, E. A., GRUENSTEIN, M. AND KOMAROV, S. A.—(1949) *J. nat. Cancer Inst.*, **10**, 255.

*Idem*, HARRIS, C. AND GRUENSTEIN, M.—(1952) *Ibid.*, **13**, 307.